# Supplementary material for: Transient Unfolding and Long-Range Interactions in Viral BCL2 M11 Enable Binding to the BECN1 BH3 Domain
Source: Biomolecules. 2020 Sep 11;10(9):1308. doi: 10.3390/biom10091308 (PMC7564285; doi:10.3390/biom10091308)
Supplement: Supplementary file 1 [file biomolecules-10-01308-s001.pdf]

Supporting Information for:
Transient Unfolding and Allosteric Interactions Enable the Binding of Viral BCL2 M11 to BECN1 BH3 Domain

Arvind Ramanathan[1,*], *Akash Parvatikar*[2], *Chakra S. Chennubhotla*[2,*], *Yang Mei*[3] *Sangita Sinha*[3,*]
[1]Data Science and Learning Division, Argonne National Laboratory, Lemont, Illinois, USA
[2]Department of Computational and Systems Biology, University of Pittsburgh, Pittsburgh, Pennsylvania, USA
[3]Department of Chemistry and Biochemistry, North Dakota State University, Fargo, North Dakota, USA
∗ **E-mail: ramanathana@ornl.gov; chakracs@pitt.edu; sangita.sinha@ndsu.edu**

Text S1: Correlation between MD simulations and NMR chemical shifts

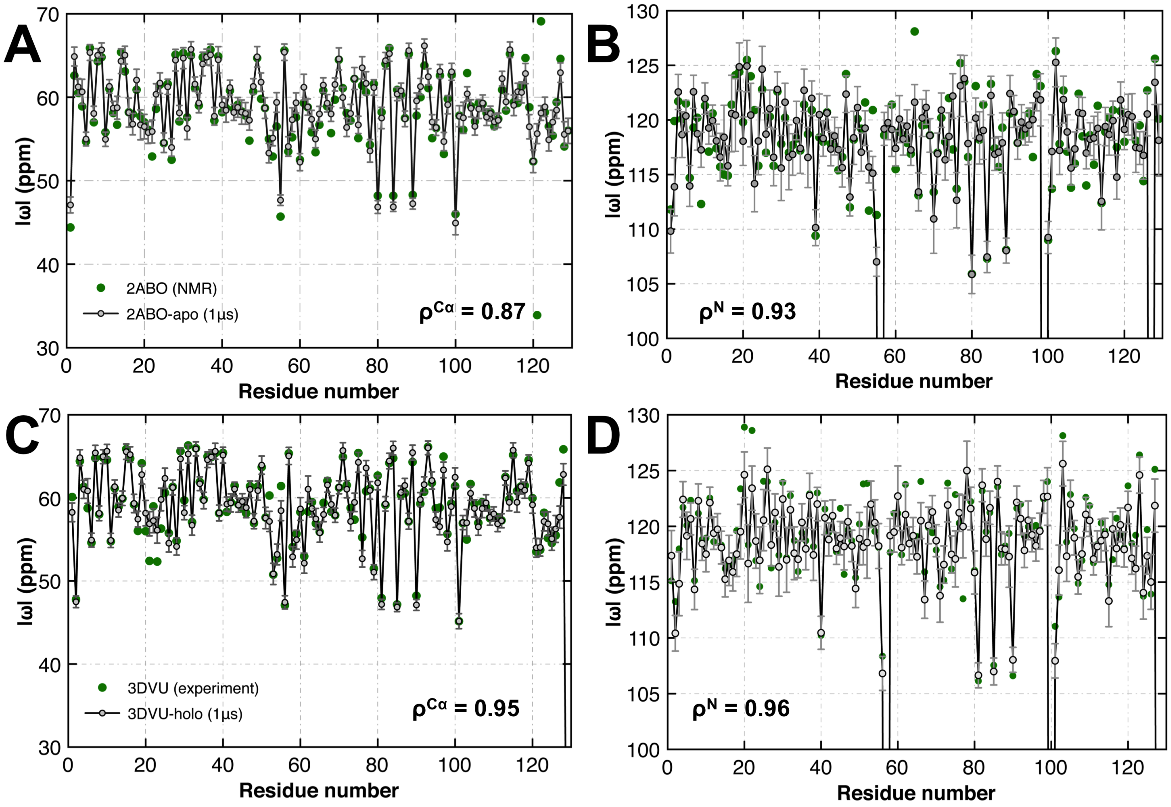

Figure S1. Chemical shift calculated from MD simulations for both apo and holo vBcl2 show good agreement with NMR determined chemical shifts. apo-vBcl2 chemical shifts of (A) C^{α} atoms with NMR indicated in green solid circles versus MD indicated in black solid lines, and (B) N atoms; (C)-(D) represent the corresponding chemical shifts for holo-vBcl2. Error bars (indicated as gray lines) show the overall variation observed from the 1 μs simulations.

In order to assess the quality of our simulations, we compared the chemical shifts determined from our MD simulations (see Methods and Materials) with experimental NMR chemical shift values. As shown in Figure S1, there is very strong correlation between the experimental NMR chemical shift values and MD-determined chemical shifts for both ^{15}N (correlation co-efficient ρ of 0.93 and 0.96 for apo- and holo- vBcl2 respectively; Figure S1A and B) and C^{α} atoms (ρ of 0.87 and 0.88 for apo- and holo- vBcl2 respectively; Figure S1C and D). Similar to previous reported studies [7], the agreement in the chemical shift values between the experimental and computational ensembles suggests that the overall quality of these simulations can be trusted for further analysis.

As an additional point of reference, we also compared the root-mean squared fluctuations (RMSF) from the X-ray determined structure (3DVU) and the MD simulations (see Supporting Figure S2) and noted a strong similarity in the fluctuations observed in the flexible regions (correlation coefficient of

0.76). Taken together, these data suggest that the MD simulations adequately capture the overall behavior of the vBcl2 both in its apo- and holo- states.

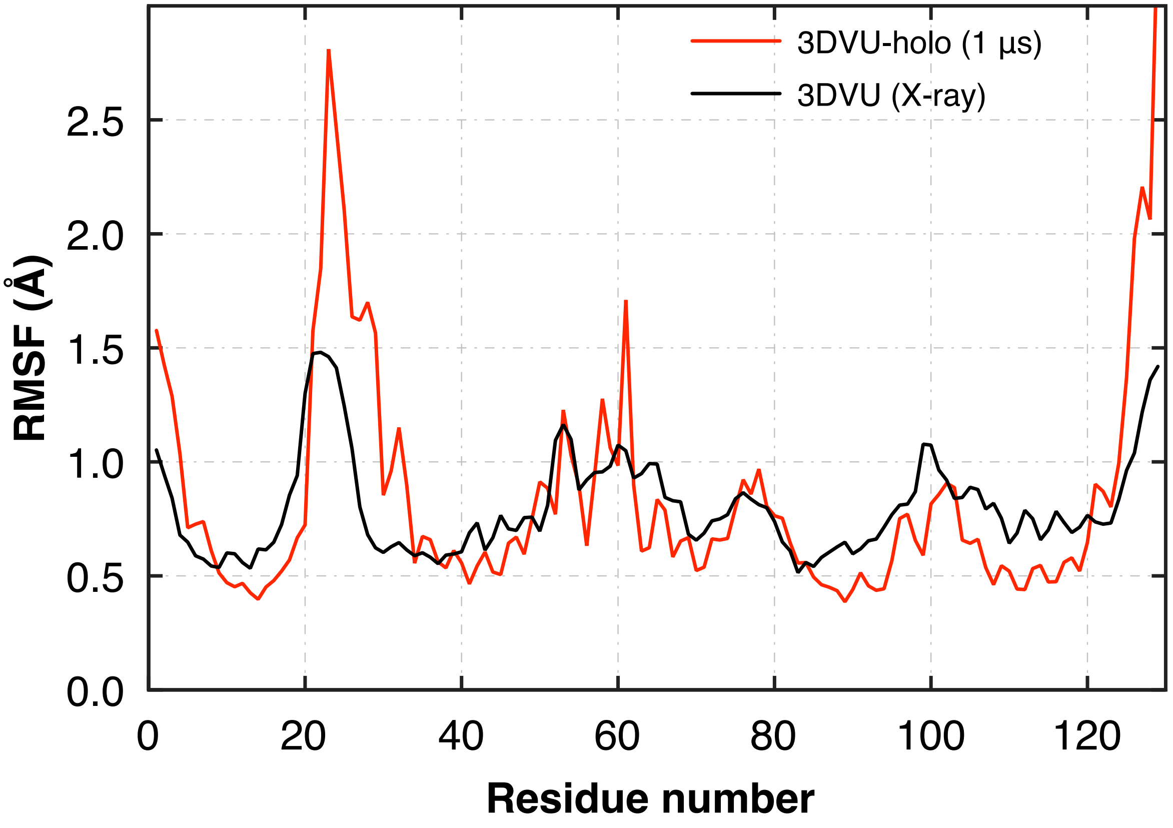

Figure S2. Chemical shift calculated from MD simulations for both apo and holo vBcl2 show good agreement with NMR determined chemical shifts. apo-vBcl2 chemical shifts of (A) C^{α} atoms with NMR indicated in green solid circles versus MD indicated in black solid lines, and (B) N atoms; (C)-(D) represent the corresponding chemical shifts for holo-vBcl2. Error bars (indicated as gray lines) show the overall variation observed from the 1 μs simulations.

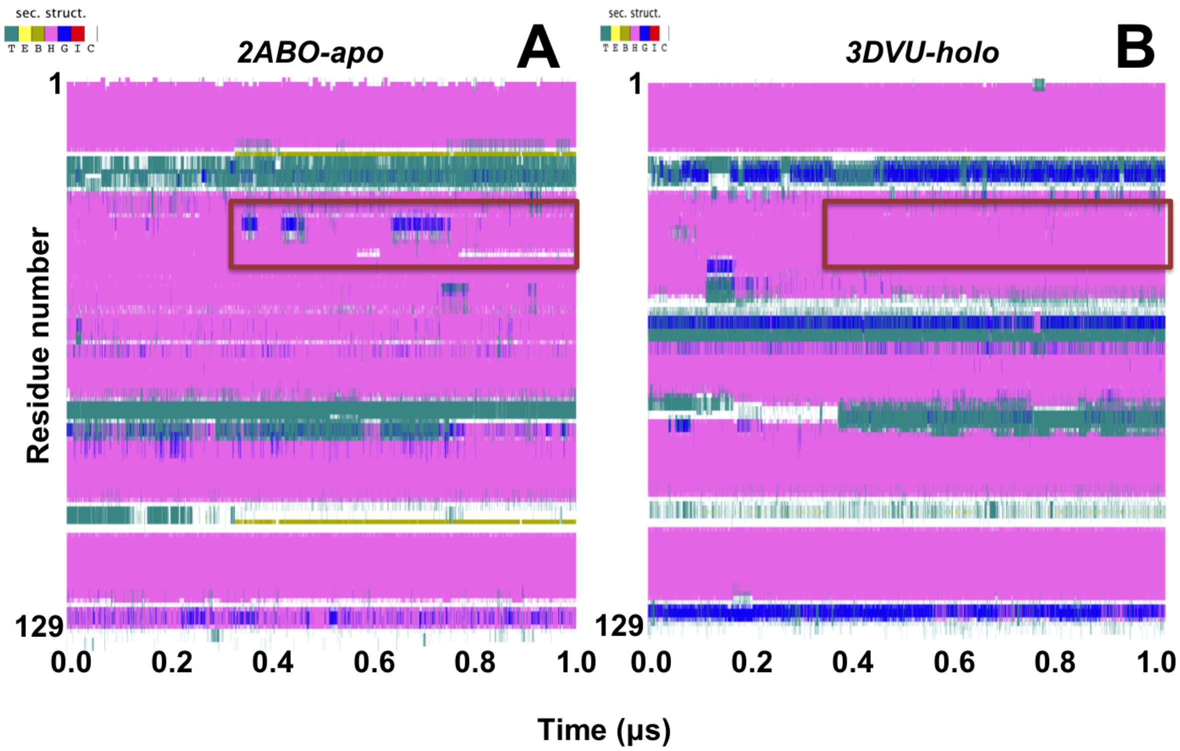

Figure S3. Partial unfolding of α in the apo-2ABO in the absence of BECN1 within the binding site. Secondary structural transitions in the microsecond time-scale (A) 2ABO-apo and (B) 3DVU-holo simulations summarized as a time-course using the DSSP plug-in within Visual Molecular Dynamics (VMD) software. The colors highlighted in the time-series are based on secondary structural content: green depicts hydrogen-bonded loops/turns; yellow highlights extended β-strands; gold depicts a residue in an isolated bridge; magenta highlights residues in α-helices; blue shows α_{3-10}-helix and white highlights coil. The red rectangle shows significant conformational transitions in α2, with coil-to-loop unfolding transitions within the core hydrophobic residues in α2.

Text S3: Analysis of binding pocket volumes in apo- and holo-M11 simulations

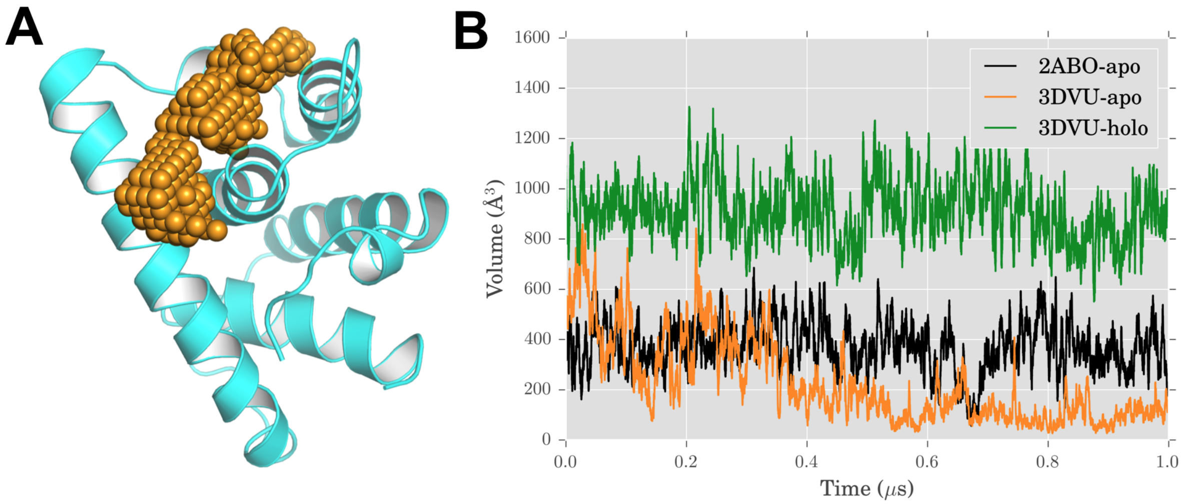

Figure S4. Binding volume of M11 BH3D binding pocket increases upon unfolding of α2. (A) The pocket selected for analysis spans the entire BH3D binding site within M11. This pocket site is depicted using orange spheres. (B) Time evolution of binding pocket volume as calculated using fpocket suite of tools. In the apo-M11 simulations (black and orange lines for 2ABO-apo and 3DVU-apo respectively) note that the volume is significantly lower compared to the holo-M11 simulations (green); however, when after partial unfolding of α2 (especially, in the last 0.4 μs of the simulation), the binding volume increases to about 600 Å^3. On the other hand, in the 3DVU-apo simulations, no such unfolding is observed, leading to a larger constriction of the binding site.

We used the fpocket [6] suite of tools to assess the volume changes for the binding cleft in our M11 simulations. The binding pocket was identified by using the PDB files for the apo-M11 (2ABO) and holo-M11 (3DVU) structures. We defined the binding pocket to consist of the overlapping spheres from the apo- and holo-M11 structures (Figure S4A). This binding pocket captures all of the regions where the BECN1 BH3D interacts with M11. Using the mdpocket plug in, we then calculated the temporal evolution of the binding site's total volume over the course of the microsecond time-scale simulations. As shown in Figure S4B, the apo-M11 simulations (both 2ABO-apo and 3DVU-apo) states show a relatively low volume ranging between 200 Å^3 and 600 Å^3. Notably, the 3DVU-apo simulation volumes are considerably lower than the 2ABO-apo simulation over the last 0.5μs, indicating a substantial packing amongst the residues in M11. Interestingly the last 0.4 μs of the 2ABO-apo simulation also shows a larger binding volume when the α2 helix undergoes partial unfolding (see Figure S3A).

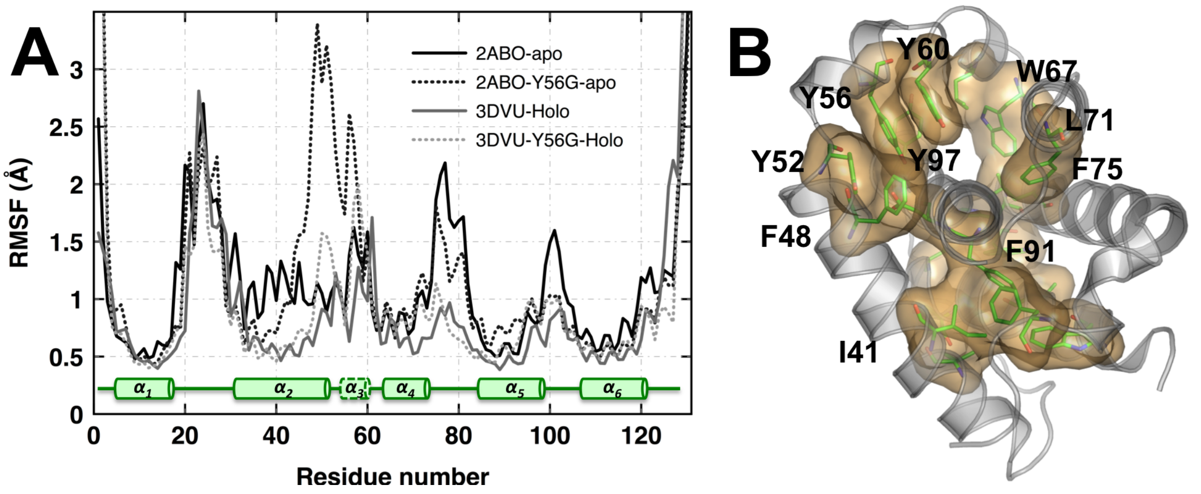

Figure S5. Mutating M11 with Tyr56Ala makes the apo-M11 more flexible, but does not affect the holo-M11 state. (A) RMSF from the Tyr56Ala simulation for the apo-M11 indicates a higher flexibility across $\alpha 2$ (dotted black line) compared to the wild type simulations (solid black line). Comparing the wild type holo-M11 (gray solid line) to the mutated holo-M11 indicates slight differences in the RMSF, but recapitulates the observed degree of fluctuations (solid gray line). The secondary structure is highlighted in green for reference. (B) The network of hydrophobic interactions is highlighted to show regions affected by the mutation.

Table S1

**M11 constructs**	K_d (**μM**)	K_a $(1/M)$	**ΔH (kJ/mol)**	**ΔG (kJ/mol)**	**ΔS (J/K . mol)**	Average distance to binding site (Å)
F79A	2.20 ± 0.03	$4.6 \pm 0.06E^6$	-36.86 ± 0.32	-31.10 ± 0.01	-20.04 ± 1.14	10.3
Y97A	1.13 ± 0.31	$0.92 \pm 0.21E^6$	-45.78 ± 3.47	-32.8 ± 0.67	-45.21 ± 12.2	12.9
L103A	2.40 ± 0.05	$4.17 \pm 0.93E^5$	-49.90 ± 12.61	-30.90 ± 0.07	-66.17 ± 43.70	20.9
M119A	1.90 ± 0.09	$5.27 \pm 0.25E^5$	-50.74 ± 3.79	-31.13 ± 0.48	-68.36 ± 14.89	16.1
L128A	0.90 ± 0.13	$1.12 \pm 0.16E^6$	-53.79 ± 1.58	-33.25 ± 0.34	-71.58 ± 6.69	16.2

Mutations to residues far away from BH3D binding site that do not substantially impact binding of the BH3D.

Text S4: Preliminary sequence and structure based analysis of viral BCL2 homologs

A pure sequence-based analysis across the vBCL2 homologs with known X-ray crystallographic structures does not provide sufficient insights into the consensus of hydrophobic interactions in the protein. As shown in the alignment, it is quite clear that the sequences share very little homology as observed from the relatively low sequence similarity profiles. Notably, many of the sequences do consist of partially conserved hydrophobic residues that are located along the sequence. However, structural analysis of the selected vBCL2s shows that these hydrophobic residues are spread around the entire 3D structure of the proteins that form this network showing a consistent pattern of interactions along α_2, α_3, α_{5-7}. It is important to note that the lengths of the $\alpha-$helices across the vBCL2s are different and therefore any sequence alignment based approach cannot fully account for such potential similarity that arises as a consequence of the structural organization.

References

1. Mika Aoyagi, Dayong Zhai, Chaofang Jin, Alexander E. Aleshin, Boguslaw Stec, John C. Reed, and Robert C. Liddington. Vaccinia virus n1l protein resembles a b cell lymphoma-2 (bcl-2) family protein. *Protein Science*, 16(1):118–124, 2007.

2. Samantha Cooray, Mohammad W. Bahar, Nicola G. A. Abrescia, Colin E. McVey, Nathan W. Bartlett, Ron A.-J. Chen, David I. Stuart, Jonathan M. Grimes, and Geoffrey L. Smith. Functional and structural studies of the vaccinia virus virulence factor n1 reveal a bcl-2-like anti-apoptotic protein. *Journal of General Virology*, 88(6):1656–1666, 2007.

3. Qiulong Huang, Andrew M. Petros, Herbert W. Virgin, Stephen W. Fesik, and Edward T. Olejniczak. Solution structure of a bcl-2 homolog from kaposi sarcoma virus. *Proceedings of the National Academy of Sciences*, 99(6):3428–3433, 2002.

4. Marc Kvansakul, Mark F. van Delft, Erinna F. Lee, Jacqueline M. Gulbis, W. Douglas Fairlie, David C.S. Huang, and Peter M. Colman. A structural viral mimic of prosurvival bcl-2: a pivotal role for sequestering proapoptotic bax and bak. *Molecular Cell*, 25(6):933 – 942, 2007.

5. Marc Kvansakul, Andrew H. Wei, Jamie I. Fletcher, Simon N. Willis, Lin Chen, Andrew W. Roberts, David C. S. Huang, and Peter M. Colman. Structural basis for apoptosis inhibition by epstein-barr virus bhrf1. *PLoS Pathog.*, 6(12):e1001236, 12 2010.

6. Vincent Le Guilloux, Peter Schmidtke, and Pierre Tuffery. Fpocket: An open source platform for ligand pocket detection. *BMC Bioinformatics*, 10(1):168, 2009.

7. Paul Robustelli, Kate A. Stafford, and III Arthur G. Palmer. Interpreting protein structural dynamics from nmr chemical shifts. *J. Am. Chem. Soc.*, 134(14):6365–6374, 2012.

8. Fabian Sievers and Desmond G. Higgins. Clustal omega for making accurate alignments of many protein sequences. *Protein Science*, 27(1):135–145, 2020/08/26 2018.

9. Andrew M. Waterhouse, James B. Procter, David M. A. Martin, Michèle Clamp, and Geoffrey J. Barton. Jalview Version 2—a multiple sequence alignment editor and analysis workbench. *Bioinformatics*, 25(9):1189–1191, 01 2009.

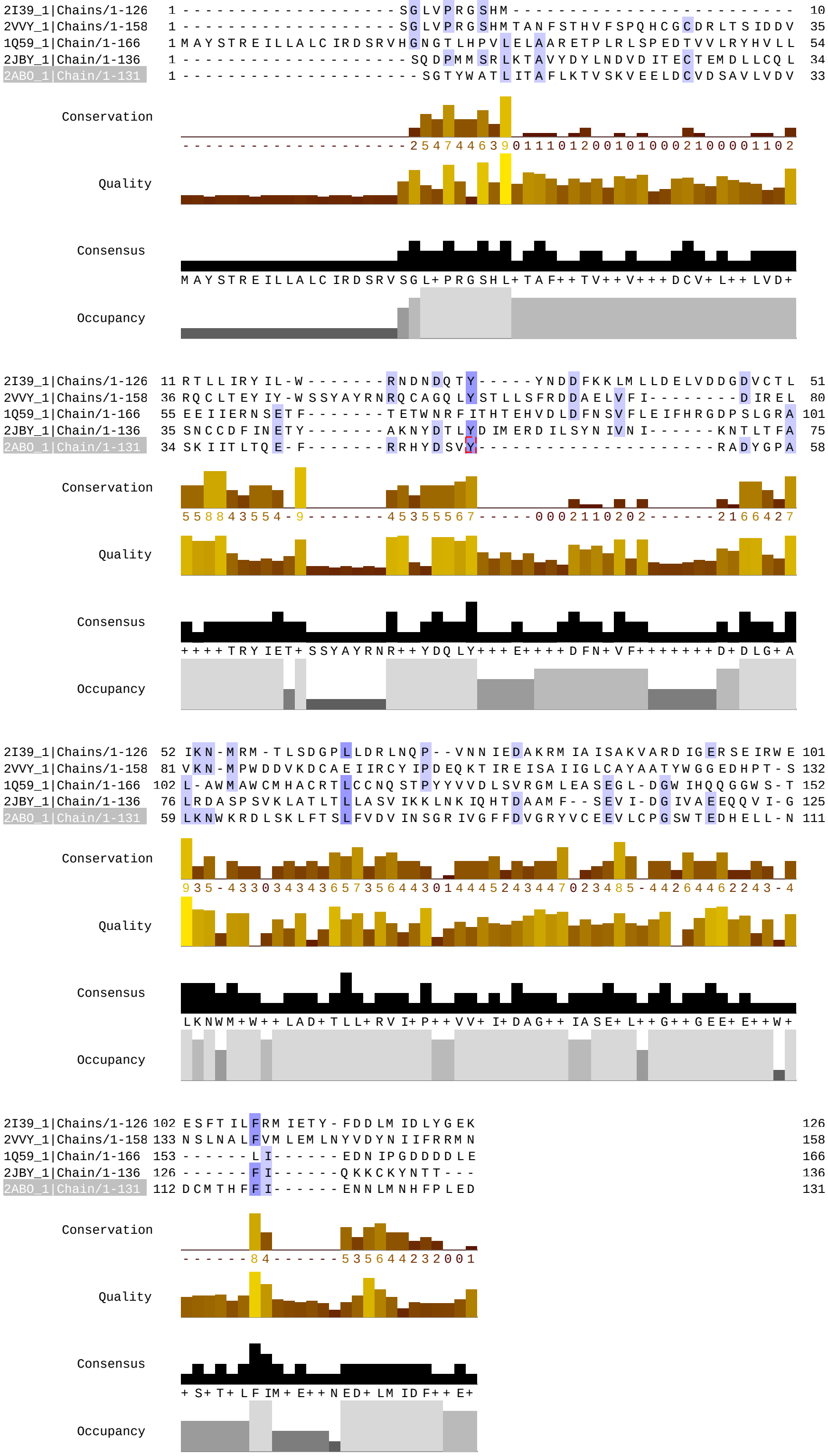

Figure S6. Sequence alignment of vBCL2s for which 3D structures are available in the PDB. The sequence of vBCL2s are poorly conserved, and meaningful alignment of sequences is further complicated by the substantial variation in the size of helices and loops comprising each protein. Despite this, some of the residues forming the allosetric hydrophobic network within M11, as identified in this study, appear to be weakly conserved across the different vBCL2s. We used Clustal Omega [8] and Jalview [9] to generate and view the sequence alignments respectively.

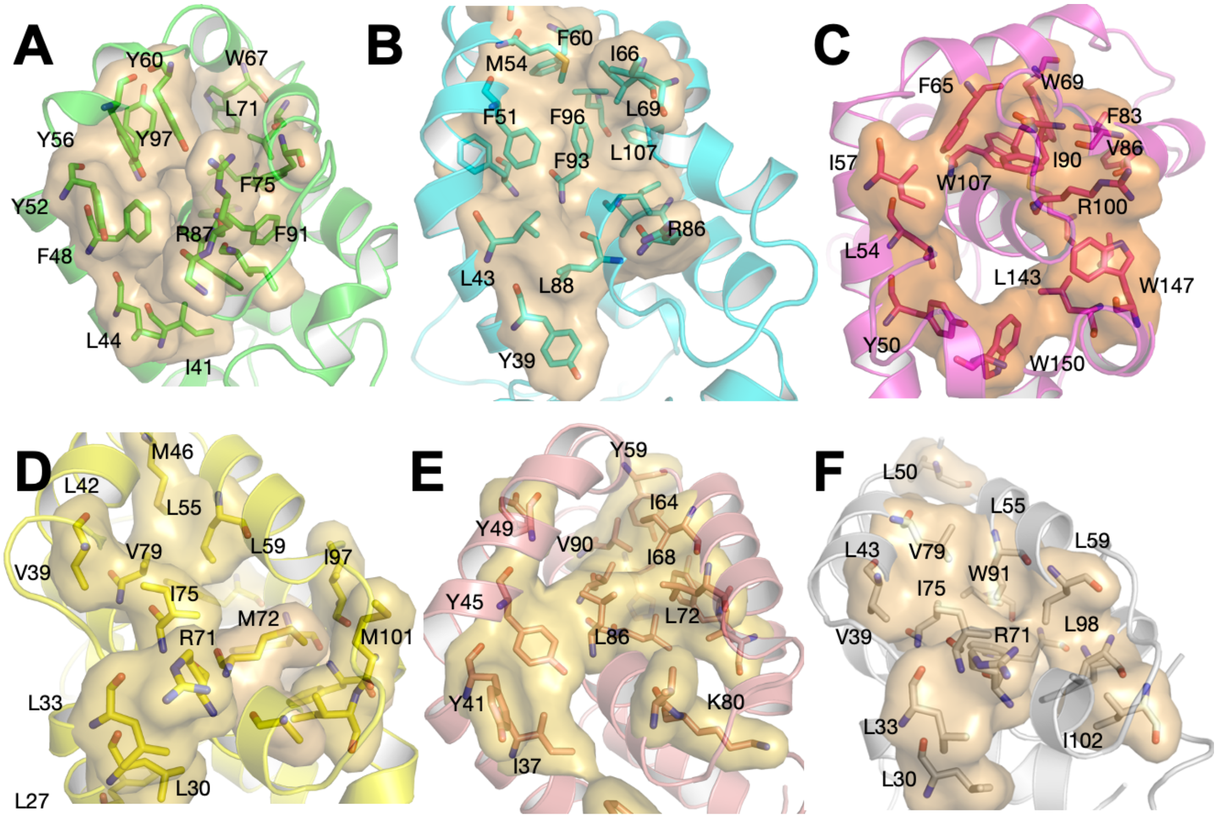

Figure S7. Structure-based sequence analysis of vBCL2 homologs reveals the presence of a network of hydrophobic interactions across each protein. (A) vBCL2 used in this study, M11 from murine γ-herpesvirus 68 viral BCL2 homolog (PDB ID 2ABO); (B) vBCL2 homolog from Kaposi's Sarcoma herpesvirus (PDB ID 1K3K [3]); (C) BHRF1 (BCL2 homolog) protein from Epstein-Barr Virus (PDB ID 1Q59 [5]); (D) Vaccinia virus N1 protein (PDB ID 2UXE [2]); (E) Myxoma virus BCL2 homolog (PDB ID 2JBY [4]) and (F) Vaccinia virus N1L protein (PDB ID 2I39 [1]). In each of these proteins, we highlight a network of hydrophobic interactions spread throughout the protein structure, that represent a consistent pattern of interactions along $\alpha 2$, $\alpha 3$, $\alpha 5-7$.
